# Exercise During Active Surveillance for Localized Low-Risk Prostate Cancer: Current Evidence and a Survivorship Framework

**DOI:** 10.3390/curroncol33100599

**Published:** 2026-10-03

**Authors:** Joseph A. Miller

**Affiliations:** Independent Scholar, Pittsboro, NC 27312, USA; docduke9@aol.com

**Keywords:** active surveillance, prostate cancer, exercise oncology, survivorship, exercise prescription, readiness monitoring, Hooper Wellness Index

## Abstract

Background: Active Surveillance (AS) is an established management strategy for appropriately selected men with localized low-risk prostate cancer, allowing many to defer or avoid definitive treatment while remaining under structured oncologic monitoring. Because surveillance may continue for years, survivorship needs during this period warrant greater attention. Objective: This narrative and conceptual review summarizes current evidence regarding structured exercise during AS and proposes a survivorship framework for individualized exercise prescription, including readiness monitoring as an emerging concept. Evidence Synthesis: Exercise-oncology evidence and AS-specific trials support benefits in cardiorespiratory fitness and selected psychosocial outcomes. A 2024 systematic review and meta-analysis of six randomized trials (332 participants) found improved VO2peak, while effects on quality of life and PSA were less certain. Recent reviews likewise conclude that exercise and other lifestyle interventions are feasible and may provide supportive benefits, but evidence remains insufficient to demonstrate consistent effects on biopsy upgrading, MRI progression, durable deferral of treatment, or long-term prostate cancer outcomes. Readiness monitoring, including potential use of the Hooper Wellness Index, has not been validated in this population and is therefore hypothesis-generating rather than established clinical practice. Conclusions: Exercise appears well positioned as one component of a broader survivorship strategy during AS. Its current value is best supported for health, fitness, function, and well-being rather than as an anticancer treatment, and it should complement rather than replace evidence-based oncologic surveillance.

## Simple Summary

Active Surveillance allows many men with localized low-risk prostate cancer to defer or avoid treatment while their cancer is carefully monitored. Because surveillance may continue for years, supporting health and quality of life during this period is also important. This narrative review examines current evidence for structured exercise among men on Active Surveillance and proposes a survivorship framework in which exercise complements, rather than replaces, oncologic monitoring. Exercise may improve fitness, physical function, and selected psychological outcomes, although current evidence does not establish that it slows prostate cancer progression. The framework emphasizes individualized exercise prescription and considers day-to-day readiness as a potential way to adjust exercise. Readiness tools such as the Hooper Wellness Index remain investigational in this population and require prospective validation.

## 1. Introduction

Active Surveillance (AS) is now recognized as the preferred initial management strategy for many men with localized low-risk prostate cancer [1], reflecting a fundamental shift from immediate treatment toward risk-adapted disease management. This approach recognizes that many low-grade prostate tumors progress slowly and may never become clinically significant [2]—that is, may not progress to disease warranting treatment or posing a meaningful threat to health—allowing carefully selected patients to defer surgery or radiation while remaining under structured observation. For purposes of this review, localized low-risk disease generally refers to clinically localized Grade Group 1 (Gleason 3 + 3) prostate cancer with PSA < 10 ng/mL and clinical stage T1–T2a, while acknowledging that contemporary surveillance criteria vary and may include selected men with favorable intermediate-risk disease [3,4]. The primary focus is structured, individualized exercise prescription in men managed with AS; observational evidence concerning general physical activity is considered complementary rather than equivalent evidence. As increasing numbers of men are managed with AS, the focus of care has expanded beyond disease monitoring alone to include strategies that preserve long-term health, functional capacity, psychological well-being, and overall survivorship.

## 2. Background

Prostate cancer remains the most frequently diagnosed non-cutaneous malignancy among men and the second leading cause of cancer-related mortality in the United States, with an estimated 333,830 new cases and 36,320 deaths projected in 2026 [5]. PSA screening has facilitated earlier detection of localized disease, and randomized evidence indicates that PSA-based screening can reduce prostate cancer mortality [6]. However, earlier diagnosis has also increased the identification of tumors that may never become clinically significant, raising concerns about overdiagnosis and overtreatment of men with favorable-risk prostate cancer [7].

Historically, many men with localized low-risk prostate cancer underwent radical prostatectomy or radiotherapy, despite having a relatively low likelihood of disease progression. Although these treatments provide excellent cancer control for appropriately selected patients, they may also result in urinary incontinence, sexual dysfunction, bowel complications, and other adverse effects that substantially influence long-term quality of life [8]. Recognition of these competing risks has led to the widespread adoption of AS as the preferred management strategy for appropriately selected prostate cancer patients [1].

Unlike most cancer management strategies, AS does not pursue immediate curative treatment. Instead, it employs structured monitoring that may include serial prostate-specific antigen (PSA) testing, digital rectal examination, multiparametric magnetic resonance imaging, and repeat biopsy or other tissue sampling at clinically appropriate intervals [9]. The frequency and combination of these assessments are individualized according to patient age, life expectancy, comorbidities, risk characteristics, previous findings, and evolving clinical guidance. Long-term randomized evidence shows low prostate cancer-specific mortality with active monitoring, surgery, or radiotherapy, while also showing higher rates of metastasis and clinical progression with active monitoring than with radical treatment [10]. These findings support careful risk selection and continued oncologic surveillance rather than implying equivalence across all disease-related outcomes.

With AS now firmly established, many men spend years and often decades living with carefully monitored prostate cancer. This evolution has naturally broadened the goals of care beyond oncologic surveillance alone. Promoting physical function, preserving sustainable health, supporting psychological well-being, and maintaining quality of life have become increasingly important components of a long-term management plan.

The prolonged duration of AS distinguishes it from many other cancer management strategies by creating an extended period during which interventions aimed at preserving health and function may meaningfully influence a patient’s lived experience.

Within this broader survivorship construct, structured exercise has emerged as one of the best- supported interventions for improving many of the physical and psychosocial challenges experienced by men with low-risk prostate cancer during prolonged surveillance.

This conceptual review examines the evolving role of exercise during AS and proposes a survivorship-oriented framework for integrating individualized exercise prescription into the contemporary management of localized low-risk prostate cancer. Particular emphasis is placed on personalized exercise programming, multidisciplinary survivorship care, and the potential role of exercise readiness monitoring as an emerging strategy to optimize enduring health and quality of life while living with prostate cancer.

## 3. Review Approach and Sources

This article was developed as a narrative and conceptual review rather than a systematic review. A targeted literature search was conducted and updated through September 2026 using PubMed/MEDLINE. Search concepts were combined around prostate cancer, Active Surveillance, exercise, physical activity, exercise oncology, survivorship, cardiorespiratory fitness, physical function, fatigue, anxiety, fear of progression, quality of life, exercise prescription, readiness, and the Hooper Wellness Index. Reference lists of relevant reviews, consensus statements, and key primary studies were also screened to identify additional clinically relevant publications.

Evidence was selected for its relevance to the central questions of the review: what is known about exercise during AS, how that evidence relates to broader cancer survivorship, and how exercise might be individualized within appropriate clinical boundaries. Direct evidence from randomized or prospective exercise studies involving men on AS was given greatest weight for AS-specific conclusions. Recent systematic reviews and meta-analyses were used to contextualize the totality and limitations of the evidence. Observational studies in broader prostate cancer populations, exercise-oncology guidelines and frameworks, and evidence from athletic readiness monitoring were used as complementary sources and were not treated as equivalent to direct AS evidence.

The review did not use a formal systematic-review protocol, duplicate screening process, quantitative meta-analysis, or formal risk-of-bias assessment. Findings were synthesized narratively according to study design, population, intervention, outcomes, and clinical relevance. The proposed application of subjective readiness monitoring represents a conceptual extrapolation from individualized exercise-prescription principles and athletic monitoring literature; it is explicitly distinguished from evidence-supported exercise recommendations and is not presented as a validated clinical protocol for men on AS.

## 4. Active Surveillance: Evolution and Current Role

Developed in response to concerns regarding overtreatment of men with low-risk prostate cancer, AS seeks to preserve the excellent oncologic outcomes associated with early detection while minimizing the treatment-related morbidity experienced by many men with indolent disease.

Evidence supporting this management approach has expanded over the past decade. Between 2014 and 2021, the proportion of eligible men managed with AS in the United States increased from approximately 27% to nearly 60% [11], reflecting substantially broader adoption in clinical practice. Consistent with these data, both the National Comprehensive Cancer Network (NCCN) and the American Urological Association (AUA) now recommend AS as the preferred initial management strategy for appropriately selected men with localized low-risk prostate cancer [3,4].

The success of AS has created opportunities that extend beyond avoiding unnecessary treatment. As prolonged surveillance has become increasingly common, preserving health, maintaining function, and supporting quality of life have become increasingly important goals of long-term management. This broader perspective has shifted attention toward survivorship strategies that complement structured oncologic surveillance while supporting patients throughout the years they live with prostate cancer.

Among these strategies, exercise has received increasing attention within contemporary survivorship care. Current guidelines recommend that exercise be incorporated into routine cancer survivorship management and tailored to each individual’s health status, treatment history, and functional capacity [12].

Although research specifically involving men undergoing AS remains relatively limited, the available direct evidence is encouraging. The Exercise During Active Surveillance for Prostate Cancer (ERASE) trial demonstrated that a 12-week program of supervised high-intensity interval training significantly improved cardiorespiratory fitness and produced short-term changes in PSA concentration, PSA velocity, and serum-stimulated prostate cancer cell growth [13]. These PSA and experimental cell-growth findings should be regarded as surrogate or exploratory biological outcomes rather than evidence of disease modification; longer-term studies are needed to determine whether exercise influences pathological progression, treatment-free survival, metastasis, or prostate cancer-specific mortality.

Additional analysis from the ERASE study reported improvements in prostate cancer-specific anxiety, fear of progression, fatigue, stress, and self-esteem [14]. A separate 2024 randomized trial of 51 men on AS found that a 16-week individualized home-based walking program improved VO2peak and reduced fear of prostate cancer recurrence and urinary obstruction/irritation compared with printed-material controls [15]. Together, these trials support short-term fitness and selected psychosocial benefits while leaving long-term oncologic effects unresolved.

Recent evidence syntheses reinforce this distinction. A 2024 systematic review and meta-analysis including six randomized trials and 332 participants found a significant improvement in VO2peak, whereas pooled effects on quality of life and PSA were less certain [16]. A 2024 scoping review reported substantial heterogeneity in physical-activity assessment and definitions of progression, with conflicting findings that precluded definitive conclusions regarding disease progression [17]. A broader 2024 systematic review and meta-analysis of nonsurgical interventions during AS similarly concluded that evidence for exercise and most other nonpharmacologic approaches remained weak [18]. More recent reviews in 2025 and 2026 have continued to characterize exercise as feasible and potentially beneficial supportive care while finding insufficient evidence for consistent effects on biopsy upgrading, MRI progression, durable deferral of definitive treatment, or long-term oncologic outcomes [19,20].

Observational evidence also provides complementary, but indirect, context. In a cohort of 2705 men with nonmetastatic prostate cancer, higher levels of vigorous physical activity after diagnosis were associated with lower prostate cancer-specific mortality [21]. Because this study was observational and was not restricted to men undergoing AS, it cannot establish causality or be interpreted as direct evidence that exercise modifies outcomes during AS.

Collectively, current evidence supports exercise as safe and beneficial for selected survivorship outcomes, particularly cardiorespiratory fitness and selected psychosocial outcomes, in men living with prostate cancer [12,13,14,15,16]. However, evidence that exercise directly delays disease progression or improves prostate cancer-specific survival during AS remains incomplete [16,17,18,19,20]. This distinction is important. The principal rationale for integrating exercise into AS is its potential to support health, function, and well-being while the disease remains appropriately monitored, not a proven ability to alter the natural history of prostate cancer.

Accordingly, structured exercise appears well positioned as one component of a broader survivorship strategy during AS. Representative evidence informing the role of exercise during AS is summarized in Table 1.

## 5. A Paradigm Shift in Active Surveillance

The widespread adoption of AS has created an opportunity to reconsider its broader purpose. While the primary objective remains the timely identification of clinically meaningful disease progression and initiation of curative treatment when indicated, the prolonged surveillance period also provides an important opportunity to implement strategies that preserve physical function, support independence, and enhance quality of life. This evolution creates an opportunity to expand AS from a strategy centered primarily on oncologic monitoring to one that also embraces the principles of comprehensive cancer survivorship. This shift is not a change in the oncologic objectives of AS, but an expansion of its clinical purpose to include the proactive promotion of long-term survivorship.

Within this broader construct, structured exercise offers one evidence-informed means of supporting health, function, and well-being during AS.

Importantly, adopting a survivorship perspective does not alter the oncologic goals of AS. Rather, it broadens the survivorship conversation surrounding it by recognizing the physical and psychological challenges men may experience during prolonged surveillance and the importance of supporting long-term health and well-being.

Broader exercise-oncology guidance supports incorporating appropriately prescribed exercise across cancer survivorship while emphasizing individualization and referral when needed [12,22]. For men undergoing AS, the extended surveillance period may provide an opportunity, when appropriate, for referral to a qualified exercise professional for individualized exercise counseling and support.

Viewed through this lens, AS remains an oncologic management strategy while also creating a prolonged period in which survivorship needs can be addressed. Individualized exercise may complement structured oncologic monitoring by supporting health, function, and quality of life during that period.

## 6. Translating the Paradigm into Clinical Practice

If AS is viewed not simply as a strategy for monitoring disease but also as a period of survivorship, the practical question becomes how men can be supported in living well while appropriate cancer surveillance continues. Exercise offers one evidence-informed means of addressing that objective. Importantly, incorporating exercise into the AS experience does not require changing the fundamental purpose or clinical structure of AS. Disease monitoring remains the responsibility of the urology and oncology team, and decisions regarding PSA testing, imaging, biopsy, and transition to definitive treatment remain within established clinical pathways. Rather, the proposed framework expands attention to the health and well-being of the individual during the potentially prolonged period in which surveillance occurs.

An important distinction should be made between general physical activity and prescribed exercise. Men on AS should be encouraged to remain physically active and reduce sedentary behavior; however, exercise represents a more structured intervention characterized by planned, purposeful, and progressive activity directed toward specific health and functional outcomes. Consequently, a recommendation to “exercise more” is not equivalent to an individualized exercise prescription. Exercise programming should consider the individual’s medical history, comorbidities, current fitness and functional capacity, previous exercise experience, symptoms, goals, preferences, and potential barriers to participation [12,23].

Implementation therefore requires an appropriate division of responsibilities. The urology or oncology team provides the relevant clinical context, identifies precautions or contraindications when present, and facilitates referral for additional evaluation when warranted. A qualified exercise professional can subsequently translate those clinical considerations into an individualized exercise program, monitor tolerance and progression, and modify the prescription as circumstances change. In practical terms, disease and treatment status establish the boundaries of the exercise prescription; readiness helps determine the appropriate dose within those boundaries on a particular day [22].

The degree of professional supervision required need not be uniform. Some men—particularly those who are medically stable, experienced with exercise, and capable of exercising safely and independently—may require relatively little ongoing supervision after appropriate assessment and instruction. Others may benefit from closer supervision because of comorbidities, low baseline fitness, unfamiliarity with exercise, functional limitations, symptoms, or lack of confidence. Supervision should therefore be viewed as a continuum rather than a fixed requirement, with the level of support adjusted as competence, confidence, and self-management capacity develop [12,22].

Reassessment and communication remain important as clinical circumstances change. New symptoms, significant changes in health status, procedures, injuries, initiation of cancer treatment, or other events that may alter exercise safety or tolerance should prompt reconsideration of the exercise prescription and, when appropriate, communication with the healthcare team. New or worsening symptoms or clinical concerns that extend beyond the exercise professional’s scope should prompt referral back to the appropriate member of the healthcare team before exercise is continued or progressed. This relationship should remain purposeful and within professional scope rather than creating an additional layer of routine medical surveillance [22].

Practical implementation must also acknowledge barriers. Access to qualified exercise professionals, cost, transportation, geographic limitations, competing responsibilities, and differences in motivation or exercise preference may influence participation. Accordingly, implementation should accommodate multiple delivery approaches, including supervised programs, community-based exercise, home-based programs, and combinations of in-person and remote support when appropriate. The objective is not to prescribe a single model of exercise delivery, but to provide sufficient structure and support for safe, sustainable participation [22].

Within this framework, exercise is not intended to replace or modify oncologic surveillance, nor should its value be judged solely by cancer-related outcomes. Rather, individualized exercise provides men with a practical means of influencing important dimensions of health, function, and well-being while living with a cancer that is being carefully monitored. In this way, exercise may complement AS by helping shift the time between surveillance visits from a period characterized primarily by observation toward an opportunity for purposeful health engagement. The proposed survivorship framework is summarized in Figure 1.

## 7. Personalizing Exercise Prescription Through Readiness Monitoring

Current exercise guidelines emphasize tailoring exercise programs to an individual’s medical history, functional capacity, treatment status, and personal goals [12,24]. However, they provide relatively little guidance regarding day-to-day modification of exercise in response to fluctuations in physical and psychological readiness. Yet these short-term variations are common among individuals living with cancer and may substantially influence exercise tolerance, safety, adherence, and targeted outcomes.

Men undergoing AS may experience fluctuations in fatigue, psychological stress, sleep quality, musculoskeletal discomfort, intercurrent illness, and other elements that may temporarily affect their ability to exercise [25]. Recognizing and responding to these daily changes represents a logical extension of personalized exercise prescription and may further enhance the safety and effectiveness of exercise oncology.

In athletic populations, several practical approaches have been developed to monitor readiness and guide daily training decisions. The Hooper Wellness Index (HWI) is a brief self-report measure incorporating sleep quality, fatigue, stress, and muscle soreness. It has been used in sports settings as a practical means of monitoring subjective wellness and training response [26].

Although the HWI has not been validated in exercise oncology populations, the underlying concept of routine wellness monitoring may have important clinical relevance for men undergoing AS for prostate cancer. A brief pre-exercise assessment could identify temporary reductions in readiness that might otherwise go undetected. This could allow exercise professionals to modify daily exercise prescriptions according to an individual’s current condition rather than relying exclusively on a predetermined approach.

Importantly, readiness monitoring should not be viewed as a reason to discourage exercise. Instead, it provides an opportunity to further individualize exercise prescription. For example, an individual reporting poor sleep, elevated stress, or excessive fatigue may benefit from temporarily reducing exercise intensity, while emphasizing mobility, flexibility, or light aerobic activity. Conversely, those demonstrating favorable readiness may be well positioned to safely progress exercise volume or intensity when appropriate.

At present, evidence supporting readiness monitoring in cancer exercise programs remains limited, and prospective studies are needed to establish its feasibility, validity, and clinical utility. Future investigation should determine whether incorporating readiness assessments into routine exercise programming improves adherence, symptom management, functional outcomes, or quality of life. If supported by future research, readiness monitoring may represent a valuable addition to individualized survivorship care for men living with localized low-risk prostate cancer during AS.

Table 2 translates these individualized exercise-prescription and readiness principles into practical clinical considerations for day-to-day application during AS.

## 8. Discussion

AS has fundamentally changed the management of low-risk prostate cancer by allowing many men to defer or avoid definitive treatment and its associated adverse effects while maintaining appropriate oncologic monitoring. Its success, however, creates an important survivorship question: how should the health and well-being of men be supported during what may become many years of surveillance?

Exercise appears well positioned as one component of that broader survivorship strategy. Available evidence supports benefits relevant to men on AS, including improvements in cardiorespiratory fitness, physical function, and selected psychosocial outcomes. Emerging studies also raise important questions regarding potential biological effects of exercise, but the current evidence is insufficient to conclude that exercise alters prostate cancer progression or long-term oncologic outcomes. The value of exercise during AS should therefore not be judged by oncologic outcomes alone [12,13,14,16,17,18,19,20].

This distinction is central to the proposed paradigm. AS appropriately focuses on monitoring the cancer; an active survivorship approach broadens attention to the individual living with it. Exercise provides one practical means through which men can influence aspects of their health, function, and well-being while remaining within established oncologic surveillance. Nutrition, sleep and recovery, stress management, and other health behaviors may similarly contribute to this broader objective, although exercise serves as the principal exemplar in the present framework.

The practical application of exercise during AS should also move beyond generic recommendations to “be active.” Individualized exercise prescription allows the type, intensity, volume, progression, and level of supervision to reflect the individual’s clinical circumstances, functional capacity, experience, goals, preferences, and changing tolerance. Within clinically appropriate boundaries, responsiveness to day-to-day readiness may provide an additional means of individualizing the exercise dose [12,23].

Readiness monitoring represents a potentially useful, but presently underdeveloped, component of this approach. Instruments such as the Hooper Wellness Index offer a simple means of capturing subjective dimensions including sleep, fatigue, stress, and muscle soreness. Although such measures have been used in athletic settings, their utility for men with prostate cancer on AS has not been established. Their application in exercise oncology should therefore be considered hypothesis-generating rather than validated clinical practice [26].

Ultimately, the proposed framework does not redefine the oncologic purpose of AS. Rather, it expands the survivorship conversation surrounding it. Successful AS can encompass both careful monitoring of the cancer and purposeful support of the health of the individual living with it. Exercise appears well positioned to contribute to that second objective while preserving the primacy of evidence-based cancer surveillance.

## 9. Limitations

Several limitations should be considered when interpreting the proposed framework. The evidence examining structured exercise specifically among men with low-risk prostate cancer managed with AS remains limited, with relatively few trials, modest sample sizes, heterogeneous interventions, and generally short follow-up periods. Consequently, evidence supporting improvements in fitness and selected patient-reported outcomes should not be extrapolated to conclude that exercise reduces disease progression, metastasis, or prostate cancer-specific mortality [16,17,18,19,20].

The proposed framework also integrates evidence and concepts drawn from the broader exercise-oncology and survivorship literature, which may not be directly generalizable to all men on AS. Individual differences in age, comorbidities, baseline fitness, socioeconomic circumstances, access to exercise services, preferences, and readiness for behavioral change may influence both participation and outcomes.

Finally, the proposed use of subjective readiness monitoring requires prospective evaluation in this population. The Hooper Wellness Index was not developed or validated specifically for cancer survivors or men on AS. Its potential role in guiding day-to-day exercise modification should therefore be considered a practical hypothesis for future investigation rather than an established standard of care [26].

## 10. Future Directions

Future research should determine how exercise can be most effectively integrated into the care of men undergoing AS. Larger and longer-duration trials are needed to establish the effects of structured exercise on cardiorespiratory fitness, strength, physical function, psychological well-being, quality of life, and other survivorship outcomes, while continuing to explore potential effects on disease-related biomarkers and long-term oncologic outcomes.

Research should also examine how exercise modality, frequency, intensity, volume, progression, and level of supervision influence adherence and outcomes. Comparative evaluation of supervised, community-based, home-based, and hybrid delivery models may help identify approaches that are both effective and scalable across diverse clinical and geographic settings.

The potential role of readiness monitoring warrants specific investigation. Prospective studies should determine whether brief subjective measures of sleep, fatigue, stress, and muscle soreness provide useful information for modifying daily exercise prescriptions and whether readiness-informed adjustments improve adherence, tolerance, safety, or longer-term outcomes.

Finally, implementation research is needed to determine how exercise professionals can be appropriately integrated with urology, oncology, primary care, and survivorship services. Such work should examine referral pathways, communication practices, accessibility, cost, professional competencies, and strategies that support increasing patient independence while maintaining appropriate connection with the healthcare team.

## 11. Conclusions

AS has become an established management strategy for appropriately selected men with low-risk prostate cancer, allowing many to defer or avoid treatment while remaining under careful oncologic monitoring. As increasing numbers of men live for extended periods on AS, attention to survivorship during surveillance becomes increasingly important.

Exercise appears well positioned as one component of a broader survivorship strategy. Although current evidence does not establish that exercise alters prostate cancer progression, its potential value extends beyond oncologic outcomes to physical fitness, function, psychological well-being, and other dimensions of health that remain important throughout surveillance.

The proposed paradigm does not seek to change what AS does well. It seeks to broaden what successful AS can mean: careful monitoring of the cancer accompanied by purposeful support of the man living with it. Within that framework, exercise offers men a practical opportunity to remain actively engaged in their health while their cancer continues to be appropriately monitored.

## Figures and Tables

**Figure 1:**
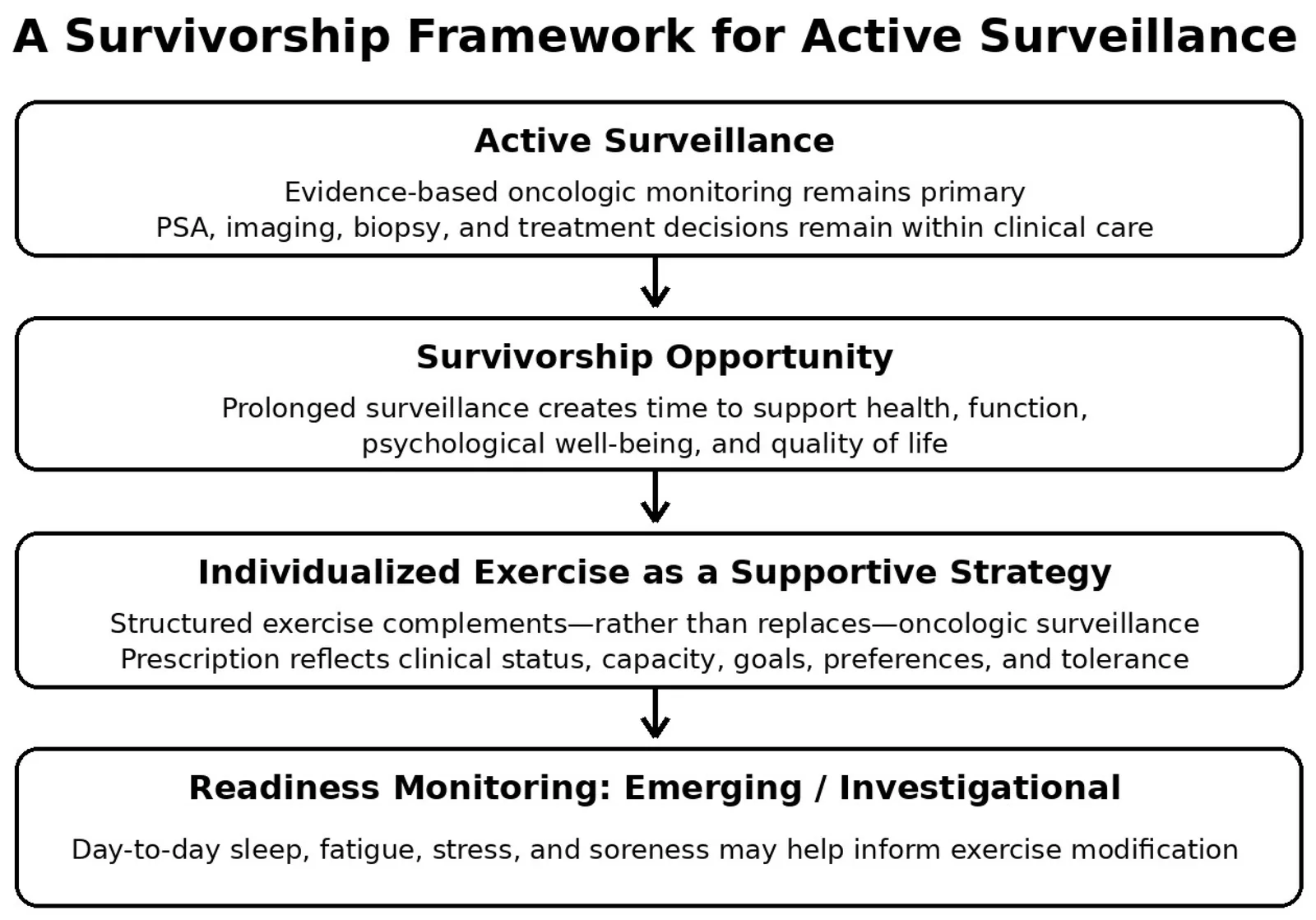
Survivorship framework for Active Surveillance in localized low-risk prostate cancer. Active Surveillance remains an oncologic monitoring strategy. Individualized exercise is presented as a supportive survivorship strategy, while readiness monitoring is an emerging, investigational approach requiring prospective validation.

**Table 1:** Representative evidence informing exercise during Active Surveillance for localized low-risk prostate cancer.

Study	Population	Exercise Intervention	Principal Findings
Kang et al. (ERASE Trial) [13]	Men with localized very low-risk to favorable intermediate-risk prostate cancer on AS (*n* = 52)	12-week, thrice-weekly supervised aerobic high-intensity interval training (HIIT)	Improved cardiorespiratory fitness; short-term reductions in PSA level, PSA velocity, and serum-stimulated LNCaP prostate cancer cell growth. Long-term clinical significance remains uncertain.
Kang et al. [14]	Men with prostate cancer on AS (*n* = 52)	12-week, thrice-weekly supervised aerobic HIIT	Improved prostate cancer-specific anxiety, fear of cancer progression, stress, fatigue, and self-esteem; quality-of-life findings were assessed but should not be generalized beyond the trial.
Kenfield et al. [21]	Men with nonmetastatic prostate cancer (*n* = 2705)	Observational assessment of leisure-time physical activity	Higher vigorous physical activity after diagnosis was associated with lower prostate cancer-specific mortality; observational evidence, not specific to AS, and not proof of causality.
Campbell et al. [12]	Cancer survivors (across tumor sites)	Exercise oncology guideline (consensus report)	Consensus evidence supports exercise for several cancer-survivorship outcomes, including physical function, fatigue, fitness, and selected quality-of-life outcomes; evidence is not specific to AS.
Van Blarigan et al. [15]	Men with low-risk prostate cancer on AS (*n* = 51)	16-week individualized home-based walking program with heart-rate monitoring and weekly exercise-physiologist support	Improved VO2peak; reduced fear of prostate cancer recurrence and urinary obstruction/irritation. Short-term trial; no evidence of long-term oncologic benefit.
Lee et al. [16]	Six randomized trials in men on AS (*n* = 332)	Systematic review and meta-analysis of six exercise/lifestyle RCTs (2–12 months)	Pooled improvement in VO2peak; quality-of-life and PSA effects less certain; larger trials needed.

**Table 2:** Practical framework for individualizing exercise prescription during Active Surveillance.

Clinical Consideration	Practical Application
Medical history	Review contraindications, comorbidities, and current medications.
Functional capacity	Assess baseline fitness, physical limitations, and prior activity level.
Exercise goals	Clarify patient priorities (e.g., improve fitness, reduce fatigue, maintain independence).
Daily readiness	Consider brief subjective assessment of sleep quality, fatigue, stress, and muscle soreness when evaluating day-to-day exercise tolerance.
Exercise modification	Adjust frequency, intensity, duration, or modality according to day-to-day readiness and clinical status.
Ongoing reassessment	Monitor response, review goals, and progress exercise as readiness and clinical status permit.

## Data Availability

No datasets were generated or analyzed during the current study.
